# Attempted suicide rates before and during the COVID-19 pandemic: interrupted time series analysis of a nationally representative sample

**DOI:** 10.1017/S0033291721004384

**Published:** 2021-10-19

**Authors:** Yael Travis-Lumer, Arad Kodesh, Yair Goldberg, Sophia Frangou, Stephen Z. Levine

**Affiliations:** 1 Faculty of Industrial Engineering and Management, Israel Institute of Technology, Haifa, Israel; 2 Department of Community Mental Health, University of Haifa, Haifa, Israel; 3 Meuhedet Health Services, Tel-Aviv, Israel; 4 Department of Psychiatry, Djavad Mowafaghian Centre for Brain Health, University of British Columbia, Vancouver, Canada; 5 Department of Psychiatry, Icahn School of Medicine at Mount Sinai, New York, NY, USA

**Keywords:** Coronavirus, epidemiology, public health policy, risk, suicide attempt, trauma

## Abstract

**Background:**

To characterize the association between the protracted biopsychosocial coronavirus disease 2019 (COVID-19) pandemic exposures and incident suicide attempt rates.

**Methods:**

Data were from a nationally representative cohort based on electronic health records from January 2013 to February 2021 (*N* = 852 233), with an interrupted time series study design. For the primary analysis, the effect of COVID-19 pandemic on incident suicide attempts warranting in-patient hospital treatment was quantified by fitting a Poisson regression and modeling the relative risk (RR) and the corresponding 95% confidence intervals (CIs). Scenarios were forecast to predict attempted suicide rates at 10 months after social mitigation strategies. Fourteen sensitivity analyses were performed to test the robustness of the results.

**Results:**

Despite the increasing trend in the unexposed interval, the interval exposed to the COVID-19 pandemic was statistically significant (*p* < 0.001) associated with a reduced RR of incident attempted suicide (RR = 0.63, 95% CI 0.52–0.78). Consistent with the primary analysis, sensitivity analysis of sociodemographic groups and methodological factors were statistically significant (*p* < 0.05). No effect modification was identified for COVID-19 lockdown intervals or COVID-19 illness status. All three forecast scenarios at 10 months projected a suicide attempt rate increase from 12.49 (7.42–21.01) to 21.38 (12.71–35.99).

**Conclusions:**

The interval exposed to the protracted mass social trauma of the COVID-19 pandemic was associated with a lower suicide attempt rate compared to the unexposed interval. However, this trend is likely to reverse 10 months after lifting social mitigation policies, underscoring the need for enhanced implementation of public health policy for suicide prevention.

## Introduction

Suicide is preventable, yet annually, approximately 800 000 people die of suicide worldwide (World Health Organization, 2014). Attempted suicide is one of the most significant risk factors for completed suicide (Bostwick, Pabbati, Geske, & McKean, 2016; Ribeiro et al., 2016). Suicide attempts are self-inflicted, injurious nonfatal behaviors with evidence of intent to die (Silverman et al., 2007). The lifetime suicide attempt rate is estimated at 1.9–8.7% and the 12-month rate at 0.2–2.0% (Nock et al., 2008). Suicide attempts are associated with extensive familial, economic, and societal burdens (Shepard, Gurewich, Lwin, Reed, & Silverman, 2016).

The coronavirus disease 2019 (COVID-19) pandemic is a complex event characterized by multiple biopsychosocial adversities experienced on an unprecedented global scale and so is termed a ‘mass social trauma’ (Feuer, 2021; Hoffman & Kruczek, 2011). Intuitive concerns have been expressed that the biopsychosocial adversities associated with the COVID-19 pandemic may increase the suicide attempt rate (Gunnell et al., 2020; Ongur, Perlis, & Goff, 2020; Reger, Stanley, & Joiner, 2020; Wasserman, Iosue, Wuestefeld, & Carli, 2020). These concerns stem from the potential increase in suicide risk factors due to COVID-19 attenuation strategies (e.g. social distancing; Reger et al., 2020) imposed on a national scale and the economic hardship introduced by the pandemic. Collectively, these factors may act as a mechanism to exacerbate loneliness and depression and, in turn, elevate the suicide attempt rate. Alternatively, it may be postulated that the COVID-19 attenuation strategies may act as a mechanism that reduces the means and opportunities for self-injurious behavior and neutralization of the impact of other risk factors (e.g. isolation) that are otherwise elevated, thus reducing the attempted suicide rate (Mann, Michel, & Auerbach, 2021). It is also possible, based on prior studies of natural disasters, partly owing to the aforementioned mechanisms, that despite distress, attempted suicide rates initially drop post-disaster. This drop has been termed a ‘honeymoon period’ (Madianos and Evi, 2010) and ‘pulling together’ phenomenon (Gordon, Bresin, Dombeck, Routledge, & Wonderlich, 2011) and likely occurs owing to social cohesion mechanisms. Following the initial honeymoon period, the suicide attempt rate is projected to increase (Kolves, Kolves, & De Leo, 2013), possibly due to adaption to and coping with a new reality (Zortea et al., 2020; Zunin & Myers, 2000).

Several large-scale epidemiological studies have compared completed suicide rates between the periods before and during the COVID-19 pandemic (John et al., 2020; Zortea et al., 2020). Most studies found that the completed suicide rate before and during the first COVID-19 pandemic wave was unchanged (Faust et al., 2021; Leske, Kolves, Crompton, Arensman, & de Leo, 2021; Pirkis et al., 2021; Vandoros, Theodorikakou, Katsadoros, Zafeiropoulou, & Kawachi, 2020) or reduced (Calderon-Anyosa & Kaufman, 2021; Qin & Mehlum, 2021; Radeloff et al., 2020; Tanaka & Okamoto, 2021). However, the suicide completion rate increased in the second wave in Japan (Tanaka & Okamoto, 2021), whereas in Maryland, USA, it reduced progressively across three waves in white but increased in black people (Bray et al., 2021).

Few observational studies scrutinized the impact of the COVID-19 pandemic on other suicidal behaviors. During the early stages of the pandemic, national self-harm rates dropped in the UK (Carr et al., 2021) and France (Jollant et al., 2020). These studies are restricted to the early pandemic phases and so may not capture the protracted effects of disruption and trauma. In addition, the effect of COVID-19 infection remains unknown. Existing evidence regarding the impact of exposure to the COVID-19 pandemic on severe suicide attempts warranting emergency room care is scarce. Therefore, there is an evidence gap with major public health implications regarding the possible impact of COVID-19 pandemic across all waves and no forecast estimates of how rates might change when the social mitigation policies are lifted.

To address these lacunae, we examined the association between exposure to three waves of the COVID-19 pandemic and the suicide attempt rate. Sensitivity analyses were implemented to consider sociodemographic factors, methodological artifacts, lockdown periods, and COVID-19 infection. Prediction forecasts were undertaken with regard to the suicide attempt rate following the termination of social mitigation.

## Methods

### Population

Under the National Health Insurance Law, healthcare services to the entire population of Israel are provided by four non-profit health maintenance organizations (HMOs; Chinitz, Shalev, Galai, & Israeli, 1998). This legislation states that all HMOs must offer nationwide services and do not differ financially or in service provision. By law, each Israeli citizen must choose to join a single HMO. HMOs cannot deny residents membership based on demographic or medical characteristics (i.e. age, location, minority-group status, and medical history). Accordingly, non-inclusion by an HMO (and hence, sample selection) would violate Israeli legislation. The current study analysis was based on data from the HMO ‘Meuhedet Healthcare Services’ (hereafter Meuhedet), which serves 14% of the total population of Israel nationwide. The source population is nationwide coverage of all Meuhedet members aged over 15 years. The study received approval from the Meuhedet-associated Helsinki Institutional Review Board with a waiver of informed consent.

### Study design

We used interrupted time series (ITS; online Supplementary eFig. 1; Bernal, Cummins, & Gasparrini, 2017, 2020; Bhaskaran, Gasparrini, Hajat, Smeeth, & Armstrong, 2013), a quasi-experimental study design (Shadish, Cook, & Campbell, 2002), widely used in COVID-19 suicide research (Leske et al., 2021; Pirkis et al., 2021), to compare monthly incident suicide attempt rates before and during the COVID-19 pandemic. Data collection covered the period from 1 January 2013 to 1 February 2021, thus included three waves of severe restrictions and extended existing studies of the first wave (Anzai, Fukui, Ito, Ito, & Takahashi, 2021; Calderon-Anyosa & Kaufman, 2021; Vandoros et al., 2020). The ITS study design used electronic health registry data that are continuously collected over time. In ITS, the data are used to identify an underlying trend before and during the COVID-19 pandemic. Hence, suicide attempt trends can be examined for distinct changes from preexisting trends, termed a counterfactual. This study design is instrumental when retrospective evaluations of population-level interventions are required.

### Outcome: suicide ascertainment

Since 2009 Meuhedet has maintained a dedicated continuous electronic health registry of severe suicide attempts. Severe suicide attempts are defined as emergency room contacts for severe self-injurious behavior requiring overnight in-patient hospitalization. Suicide attempts in the registry are included based on a specific internal code (determined by the emergency staff arriving at the scene and the clinical evaluation of the emergency room staff) conveyed from the emergency room to the Ministry of Health, then to Meuhedet. In the current study, over the entire study period, incident suicide attempts were scrutinized. Namely, the monthly severe suicide attempt incident rate (suicide attempts, hereafter) was computed by dividing the monthly count of incident suicide attempts by the number of insured members.

### Exposure to COVID-19

The interval from 1 January 2013 to 1 February 2020 was classified as the ‘unexposed’ period. The first reported confirmed case of COVID-19 in Israel was on 27 February 2020, and the first lockdown started on 14 March 2020. Hence, we classified the interval starting on 1 March 2020 and ending on 1 February 2021 as the ‘exposed’ period. For clarity, we summarized the COVID-19 pandemic policy restrictions in Israel during the study period (online Supplementary eTable 1).

### Covariates

The covariates considered were a time vector based on a sequence of months from 1 January 2013 to 1 February 2021, the COVID-19 pandemic binary indicator of ‘exposure’ and their interaction. Additional covariates include an offset term to model event rates and seasonal Fourier terms to model the seasonal factors.

### Statistical analysis

For the primary analysis, we fitted a Poisson regression model and quantified the relative risk (RR) and the associated 95% confidence intervals (CIs) of the total monthly incident suicide attempt rate to scrutinize the exposure effect. Specifically, the ITS analysis implemented the model with time (as a monthly sequence during the entire 7-year period of the study period), exposure period (i.e. unexposed or exposed), and the time-exposure period interaction. Additional covariates were an offset term to model event rates and seasonal Fourier terms to model the seasonal factors.

We extended the Poisson regression model of the primary analysis to forecast future suicide incident rates. We modeled three forecast scenarios for the period starting on 1 March 2021 and ending on 1 December 2021. These forecast scenarios: (1) assumed no ongoing effects of the COVID-19 pandemic; (2) assumed ongoing effects of the COVID-19 pandemic; and (3) were based on the intervals before and during the COVID-19 pandemic.

The robustness of the primary analysis results was challenged in a series of 16 sensitivity analyses addressing groups with different socioeconomic characteristics, methodological artifacts, and COVID-19 pandemic-related public health considerations of lockdown and infection. First, we conducted eight separate sensitivity analyses focusing on sociodemographic characteristics known to influence attempted suicide, and specifically by: sex across all age-groups, sex for working-age groups, and for low, medium and high socioeconomic status (SES; see online Supplementary eText 1), and for all persons aged over 65.

Second, we tested the potential influence of methodological factors by examining different seasonal decompositions, under-over dispersion of suicide attempts, and 15-day intervals to scrutinize the potential artifact of aggregation. Next, we used a negative-control group study design and compared the attempted suicide rates in Israel during the COVID-19 pandemic with those during the Gaza war. This war lasted from 8 July 2014 to 26 August 2014, cost the lives of soldiers and civilians, and exposed the civilian population to rocket fire, and so maybe considered traumatic.

Third, we conducted three analyses to consider COVID-19 pandemic-related factors. We tested for potential differences in suicide incident rates during the lockdown-on and lockdown-off periods based on 15-day intervals. Next, we implemented an ITS analysis like the primary analysis but restricted the pandemic exposure period to persons without COVID-19 infection. Finally, we compared the attempted suicide rates of the COVID-19 positive with negative cases during the COVID-19 pandemic exposure period with a test for equality of proportions (Hogg, Tanis, & Zimmerman, 2013).

Analyses were implemented in R (R Core Team, 2020) with the packages tsModel (Peng & McDermott, 2013) and ggplot2 (Wickham, 2016).

## Results

The study sample consisted of 852 233 individuals (male: *N* = 424 240, 49.8%; female: *N* = 427 993, 50.2%), with a total suicide incident count of 1148 cases (total cumulative rate per 100 000 = 134.7, 95% CI 127.02–142.73) across time. The monthly suicide incidence across the study intervals ranged from 4 to 21, with an average of 11.71 (s.d. = 4.44), and ranged from 0.52 to 2.52 suicides per 100 000 in the population.

**Fig. 1:**
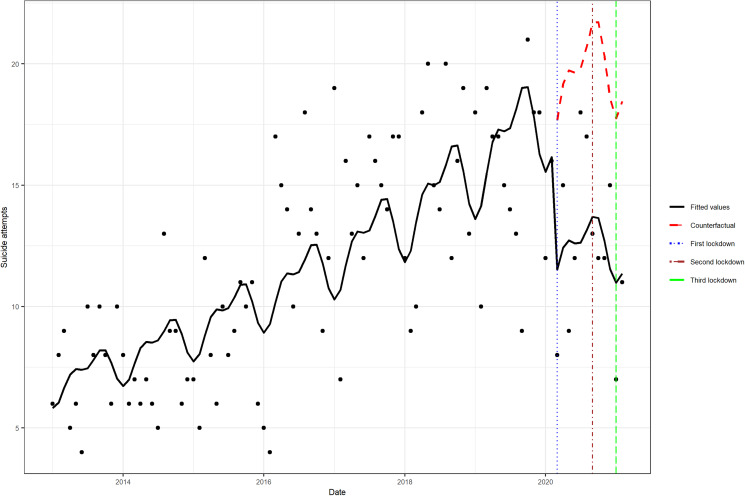
Comparison of the periods with and without COVID-19 pandemic exposure. The counterfactual refers to the predicted values had no COVID-19 pandemic occurred, and the fitted values are estimated based on the Poisson regression model.

In the unexposed period (1 January 2013 to 1 February 2020), the attempted suicide incidence rates increased linearly with time and, as observed by the counterfactual, were expected to increase further had the COVID-19 pandemic not occurred (Fig. 1). In comparison, the exposed interval (1 March 2020 to 1 February 2021) was associated with a lower incident rate of attempted suicide. The exposed interval showed a statistically significant (*p* < 0.05) decrease in the RR of attempted suicide incidence (RR = 0.63, 95% CI 0.52–0.78). The model assumptions were not violated by residual autocorrelation and residual partial autocorrelations (online Supplementary eFig. 2).

**Fig. 2:**
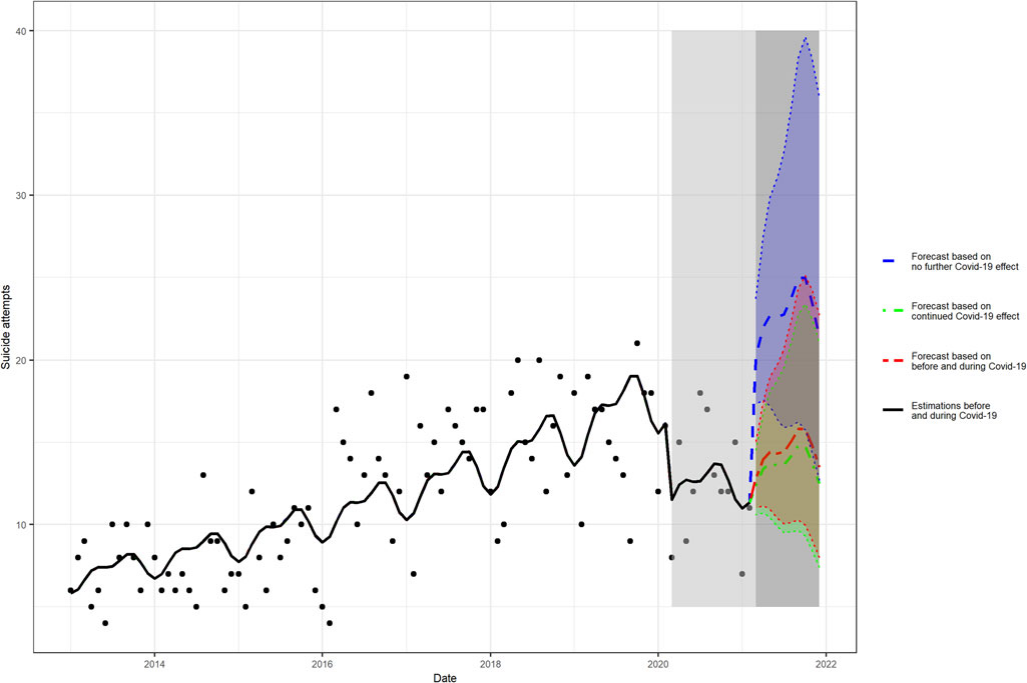
Three scenarios of forecasted COVID-19 pandemic effects on suicide attempts at 10 months. The following three scenarios (from 1 March 2021 to 1 December 2021) were scrutinized (1) assuming no ongoing effects of the COVID-19 pandemic; (2) assuming ongoing effects of the COVID-19 pandemic; and (3) based on the intervals before and during COVID-19 pandemic.

Forecasted suicide attempt rates at 10-month following the end of social mitigation were scrutinized based on three different scenarios (Fig. 2; online Supplementary eTable 2). These results show that without the seasonal component, all figures display a monotonic increase in the forecast interval. Such fluctuations in the forecasts occur due to the seasonal pattern. The forecasted incident suicide rates at 10 months were estimated at 21.38, 95% prediction interval (PI) (12.71–35.99) assuming no ongoing pandemic effect, and 12.49, 95% PI (7.42–21.01) assuming an ongoing pandemic effect. The third scenario was based on the data from the intervals before and during the COVID-19 pandemic exposure and yielded a forecast incident suicide rate of 13.50, 95% PI (8.01–22.74). Of note, for comparison, the median suicide attempt rate of the unexposed period was 11.5.

### Sensitivity analyses

Sensitivity analyses were undertaken to consider groups with potentially differential suicide attempt risks based on their sociodemographic characteristics (online Supplementary eTable 3). The results of the primary analysis replicated in a series of sensitivity analyses restricted to groups of males and females across all ages (online Supplementary eFig. 3), males and females of working age (online Supplementary eFig. 4), low and medium SES groups (online Supplementary eFig. 5), and among persons aged 65 and older (online Supplementary eFig. 6). The COVID-19 pandemic had a null effect on the RR of attempted suicide among persons with high SES (online Supplementary eTable 3). We did not identify modification by statistical artifacts of seasonal adjustment (online Supplementary eFig. 7) or under- or over-dispersion of suicide attempts (online Supplementary eFig. 8). Altering the underlying time scale to 15-day intervals (online Supplementary eFig. 9) produced identical point-precision estimates to the primary analysis. Comparison with the period of the Gaza war (online Supplementary eFig. 10), yielded comparable point-precision estimates to the primary analysis. However, although suicide attempts reduced during the COVID-19 pandemic, they increased during the Gaza (online Supplementary eFig. 10).

Lockdown on and off periods did not differ in the incidence of severe suicide attempts (online Supplementary eFig. 11). Next, we examined the entire study period and restricted the exposed period to intervals with COVID-19 negative cases, and observed a statistically significant (*p* < 0.05) reduced RR with comparable point estimates to the primary analysis (online Supplementary eTable 3, eFig. 12). Comparison of suicide rates between COVID-19-positive and COVID-19-negative cases for the exposure period showed a null (*p* > 0.05) difference in proportions between the two groups [difference = 10^−5^ (0.36), 95% CI 10^−5^ (−2.54 to 3.26), *p*-value = 1.00].

## Discussion

We leveraged epidemiological data implemented a quasi-experimental design with national coverage spanning seven years before the COVID-19 pandemic, a year of the COVID-19 pandemic, information about the three lockdown intervals, and COVID-19 infection status to test for an effect on the rate of severe attempted suicide. Despite the increasing time trend during the unexposed period before the pandemic, we observed that the suicide incidence rate statistically significantly dropped during the COVID-19 pandemic. This conclusion was reinforced by 14 of 15 rigorous sensitivity analyses that scrutinized the suicide attempt rate in diverse sociodemographic groups, accounted for methodological artifacts, considered lockdowns and COVID-19 infection status. Forecasting indicated that following the lifting of COVID-19 pandemic-related social restrictions, the rate of severe suicide attempts will likely increase over a 10-month period.

The current study is the first to examine severe suicide attempts during the protracted COVID-19 pandemic intervals, including consideration of three lockdowns and COVID-19 infection status. The result of the primary analysis, reinforced by sensitivity analyses, generally aligns with the published studies that focused on the early phases of the pandemic and showed reduced rates of completed suicide (Calderon-Anyosa & Kaufman, 2021; Qin & Mehlum, 2021; Radeloff et al., 2020; Tanaka & Okamoto, 2021) and self-harm (Carr et al., 2021; Jollant et al., 2020). Notably, in our study, the monthly suicide rate did not differ between COVID-19-positive cases and those not infected. Possibly, the lockdown effect was negligible because socioeconomic and psychological adversity continued to be present throughout the COVID-19 period.

There are several potential explanations for the reduction in pandemic-related attempted suicides. Movement restrictions may have reduced opportunities for suicide attempts. Possibly, these acted as a means-restriction mechanism that neutralized the impact of other risk factors (e.g. loneliness) that would otherwise be heightened during the COVID-19 pandemic (Mann et al., 2021). Alternatively, psychological responses to the COVID-19 pandemic may follow the disaster model (Zunin & Myers, 2000). According to this model, generally, individuals display positive affect during the initial phase of a disaster, focusing less on themselves and more on the disaster response (Zunin & Myers, 2000). Hence, the early disaster period is usually associated with high levels of social cohesion. The post-disaster is usually characterized by a ‘honeymoon period’ of general relief, followed by negative emotional states associated with disappointment in the pace of disaster recovery and the ‘new normal’ conditions of life. The disaster model perspective appears to provide the most parsimonious explanation for the current findings concerning both the observed and the forecast suicide attempt rates.

Groups of males, females, as well as working-age males and females, were scrutinized in sensitivity analyses. In the current results, all of the groups were characterized by comparable reduced RR during the COVID-19 pandemic (evidenced by the overlapping 95% CIs; online Supplementary eTable 3). Generally, however, the suicide attempt rate was higher among females than males in our results. This result is consistent with suicide research demonstrating higher attempted suicide rates among females compared to males (Canetto & Sakinofsky, 1998; Miranda-Mendizabal et al., 2019).

Notably, we include three forecast scenarios to anticipate service needs after the end of the period of pandemic-related social restrictions. All of the three 10-month forecast scenarios scrutinized projected that the suicide attempt rates would increase. This suggests that under conditions of the lifting of restrictions, it appears likely that the forecasted increase in the suicide attempt rate after 10 months is most likely.

## Limitations

There are at least six issues that deserve consideration. First, it is debatable as to whether causal inference is possible given the current study design. On the one hand, it is not possible to eliminate confounders (e.g. occupations at risk), and there is no control group which makes causal inference difficult. On the other hand, we used an ITS that is a strong quasi-experimental design (Grosz, Rohrer, & Thoemmes, 2020; Shadish et al., 2002), the analysis of groups with differential suicide attempt risks did generally not attenuate the primary study result, and an empirical experiment of attempted suicide would be unethical (Shadish et al., 2002).

Second, circumstances specific to Israel may have contributed to our results. For instance, compared to most nations, in the first wave, Israel experienced low COVID-19 mortality yet a relatively high number of COVID-19 cases and entered the second wave and lockdown sooner (Greener, 2021). Hence, caution is warranted in extrapolating our results to other nations. Nonetheless, this limitation is balanced by the findings of three waves of COVID-19 and COVID-19 infection status, different forecast scenarios, and evidence pointing to generally comparable COVID-19-related suicide rates across nations (Pirkis et al., 2021). Religious and ethnic groups in Israel have different suicide rates (Brunstein Klomek et al., 2016; Gal et al., 2012; Levinson, Haklai, Stein, Polakiewicz, & Levav, 2007). For example, attempted suicide rates among men were lower for Muslims compared to Jews (Gal et al., 2012). In the data source of the current study, we lack information on religious affiliation and ethnicity, so these cannot be ruled out as a source of confounding. Nonetheless, generally, our results replicated subgroups with differential risks. Furthermore, our study builds on the multiple studies demonstrating that Israel is an ideal setting to study all types of effects of COVID-19 (Bar-On et al., 2021; Shahar et al., 2021).

Third, the study data are ecological and lack detailed individual-level information that may have offered a more fine-grained perspective. For example, although both the COVID-19 pandemic and the Gaza war exposure periods contribute separately to changes in suicide rates, it is not possible to estimate an interaction term between the two exposures as both occurred at different, non-overlapping ecological intervals based on time-series data. Future research should ascertain individual-level data to address this issue directly.

Fourth, the width of the 95% CIs for the RR is reasonable for the primary analysis and most sensitivity analyses (i.e. width of approximately 0.3, online Supplementary eTable 3), with notable exceptions, such as age 65 plus and high SES. Overall, the width of the CIs is affected by the relatively small sample size for the exposure period on which the RR is based. Also, all the CIs, except for the high SES group, do not contain the value of 1, corresponding to a statistically significant (*p* < 0.05) finding. Therefore, we can be reasonably confident in the estimates of the primary analysis and most but not all of the sensitivity analysis.

Fifth, our data were restricted to the third-largest HMO in Israel, covering 14% of the Israeli population; hence the use of other HMO data may have given different results. However, selection by an HMO would be illegal, no other HMO has a dedicated suicide attempt registry, and we implemented multiple sensitivity analyses to examine select subgroups. Accordingly, although other results may have emerged, the current HMO study data are the best to study suicide attempts in Israel.

Sixth, inferences regarding our conclusions are restricted to severe suicide attempts. The number of severe suicide attempts may be under-estimated, a form of miss-classification which makes the comparison more stringent than otherwise.

Offsetting our study limitations, we used a nationally representative cohort with minimal missing data and robust sensitivity analyses. Selection and attrition biases are unlikely to explain our findings.

In sum, in the results of this national study, we observe that severe suicide attempt trends increased before the pandemic and dropped during the pandemic and offer a warning of the potential increase in suicide attempts in the months following the offset of pandemic-related social restrictions.

## Supplementary material

For supplementary material accompanying this paper visit http://dx.doi.org/10.1017/S0033291721004384. click here to view supplementary material
