# Supplementary material for: Attempted suicide rates before and during the COVID-19 pandemic: interrupted time series analysis of a nationally representative sample
Source: Psychol Med. 2021 Oct 19;53(6):2485–91. doi: 10.1017/S0033291721004384 (PMC8564043; doi:10.1017/S0033291721004384)
Supplement: Supplementary file 1 [file S0033291721004384sup001.doc]

**Attempted suicide rates before and during the Covid-19 pandemic: Interrupted time series analysis of a nationally representative sample**

Yael Travis-Lumer, Arad Kodesh, MD, Prof. Yair Goldberg, PhD, Sophia Frangou, MD, PhD, Stephen Z. Levine PhD

[eText 1: Socioeconomic status 2](#__RefHeading___Toc84432229)

[eTable 1 Covid-19 Israel Policy Restrictions 3](#__RefHeading___Toc84432230)

[eTable 2 Forecasted Values and 95% Prediction Intervals, for all Three Scenarios 4](#__RefHeading___Toc84432231)

[eTable 3 Relative Risks (RR), the Associated 95% Confidence Intervals (CI), P Values, and Total Suicide Attempt Rates per 100,000 5](#__RefHeading___Toc84432232)

[eFigure 1 a Basic ITS Design 6](#__RefHeading___Toc84432233)

[eFigure 2 Residual Autocorrelation - Primary Poisson Model 7](#__RefHeading___Toc84432234)

[eFigure 3 Scatterplot and Regression Fitted Values for Males then Females 8](#__RefHeading___Toc84432235)

[eFigure 4 Scatterplot and Regression Fitted Values for Males of Working Age and Females of Working Age 9](#__RefHeading___Toc84432236)

[eFigure 5 Scatterplot and Regression Fitted Values for Different SES Groups 10](#__RefHeading___Toc84432237)

[eFigure 6 Scatterplot and Regression Fitted Values for Adults aged over 65 11](#__RefHeading___Toc84432238)

[eFigure 7 Scatterplot and Regression Fitted Values for STL and MA Seasonal Decompositions 12](#__RefHeading___Toc84432239)

[eFigure 8 Scatterplot and Regression Fitted Values for Quasi-Poisson Regression 13](#__RefHeading___Toc84432240)

[eFigure 9 Scatterplot and Regression Fitted Values for 15 Day Intervals 14](#__RefHeading___Toc84432241)

[eFigure 10 Scatterplot and Regression Fitted Values for Covid-19 and 2014 Gaza War 15](#__RefHeading___Toc84432242)

[eFigure 11 Scatterplot and Regression Fitted Values for 15 Day Intervals and Covid-19 + Lockdown 16](#__RefHeading___Toc84432243)

[eFigure 12 Scatterplot and Regression Fitted Values restricted to non Covid-19 Cases 17](#__RefHeading___Toc84432244)

[eReferences 18](#__RefHeading___Toc84432245)

# eText 1: Socioeconomic status

The demographic covariates were birth date, sex, and city of domicile. Using the city of domicile, we used a neighborhood SES measure developed and validated by the Israeli Central Bureau of Statistics (Central Bureau of Statistics, 1995). This SES measure was used in many studies, including schizophrenia research (Goldberg et al., 2011; Levine, Levav, Goldberg, et al., 2016; Levine, Levav, Yoffe, et al., 2016). The nation is classified into 1,559 “geographical units,” each of which is an area with approximately 2,000 residents. Each area is as homogeneous as possible regarding ethnic background, culture, electronic appliances, and income. Villages with less than 2,000 residents are unclassified to protect anonymity, although such small areas are found to be protective against adverse mental health outcomes, perhaps owing to social cohesion. Each area is given a ranking from 1 to 20 based on information on a wide range of factors, including demographic information, education, the standard of living, employment, and social benefits (Central Bureau of Statistics, 1995).

# eTable 1 Covid-19 Israel Policy Restrictions

| Interval | Date begins | Date ends | Restrictions |
| --- | --- | --- | --- |
| Pre-lockdown 1 | 2020-03-01 | 2020-03-13 | International travel banned |
| Lockdown 1 | 2020-03-14 | 2020-04-30 | All schools closed, workplaces closed, restrictions on gatherings, stay at home, internal movement restrictions, international travel banned |
| Post-lockdown 1 | 2020-05-01 | 2020-09-18 | Restrictions on gatherings, stay at home, internal movement restrictions, international travel banned |
| Lockdown 2 | 2020-09-18 | 2020-10-17 | All schools closed, workplaces closed, restrictions on gatherings, stay at home, internal movement restrictions |
| Post-lockdown 2 | 2020-10-18 | 2020-12-26 | All schools closed, restrictions on gatherings, stay at home, internal movement restrictions, international travel banned |
| Lockdown 3 | 2020-12-27 | 2021-02-07 | All schools closed, workplaces closed, restrictions on gatherings, stay at home, internal movement restrictions, international travel banned |
| Entire Covid-19 period | 2020-03-01 | 2021-02-28 | Public events canceled |

Note. Based on the Oxford Covid-19 we identified nation-wide implemented Covid-19 attenuation strategies in Israel (Hale et al., 2021). All schools closed except nurseries. Workplaces closed for all but essential workplaces. For each interval and each restriction, we compared the mode value of the restriction to a threshold to classify whether the restriction occurred during the interval. For most restrictions, we used the Oxford Covid-19 Government Response Tracker threshold value of two, where for international travel ban, restrictions on gatherings, and workplaces closed, we used a threshold value of three.

# eTable 2 Forecasted Values and 95% Prediction Intervals, for all Three Scenarios

| Time | Scenario 1 | Scenario 2 | Scenario 3 |
| --- | --- | --- | --- |
| March 2021 | 20.28 (17.32, 23.74) | 12.41 (10.60, 14.53) | 12.86 (10.98, 15.06) |
| April 2021 | 22.00 (17.58, 27.52) | 13.39 (10.71, 16.76) | 13.97 (11.17, 17.49) |
| May 2021 | 22.67 (17.21, 29.87) | 13.73 (10.43, 18.09) | 14.41 (10.93, 18.99) |
| June 2021 | 22.58 (16.39, 31.09) | 13.6 (9.88, 18.73) | 14.32 (10.40, 19.74) |
| July 2021 | 22.76 (15.89, 32.61) | 13.65 (9.53, 19.55) | 14.42 (10.06, 20.68) |
| August 2021 | 23.78 (16.01, 35.32) | 14.18 (9.55, 21.06) | 15.06 (10.13, 22.38) |
| September 2021 | 24.97 (16.25, 38.36) | 14.81 (9.64, 22.76) | 15.81 (10.28, 24.31) |
| October 2021 | 24.99 (15.76, 39.64) | 14.75 (9.30, 23.39) | 15.82 (9.97, 25.11) |
| November 2021 | 23.40 (14.32, 38.26) | 13.74 (8.4, 22.46) | 14.79 (9.04, 24.21) |
| December 2021 | 21.38 (12.71, 35.99) | 12.49 (7.42, 21.01) | 13.50 (8.01, 22.74) |

Note. Three forecasts from 1 March 2020 2021 to 1 December 2021. Abbreviations Scenario (1) no ongoing effects of the Covid-19 pandemic; (2) ongoing effects of the Covid-19 pandemic; and (3) based on the intervals before and during Covid-19 pandemic. As a reference, forecasts for December 2021 may be contrasted to past observed values for December 2019 (18) and December 2020 (15). Multi-step prediction intervals (PIs) were calculated using the multi-step forecast standard deviation for the drift method (Hyndman & Khandakar, 2008).

# eTable 3 Relative Risks (RR), the Associated 95% Confidence Intervals (CI), P Values, and Total Suicide Attempt Rates per 100,000

| Model | RR (95% CI) | P value | Suicide rate (95% CI) |
| --- | --- | --- | --- |
| Primary Poisson | 0.63 (0.52, 0.78) | P<0.001 | 134.70 (127.02, 142.73) |
| Males | 0.54 (0.39, 0.75) | P<0.001 | 108.19 (98.52, 118.56) |
| Females | 0.70 (0.54, 0.90) | 0.01 | 160.98 (149.19, 173.47) |
| Working Males | 0.54 (0.39, 0.76) | P<0.001 | 114.71 (104.08, 126.14) |
| Working Females | 0.76 (0.58, 1.00) | 0.05 | 172.61 (159.39, 186.63) |
| Low SES | 0.56 (0.37, 0.86) | 0.01 | 104.88 (91.79, 119.31) |
| Medium SES | 0.62 (0.48, 0.81) | P<0.001 | 151.52 (140.69, 162.97) |
| High SES | 0.73 (0.42, 1.27) | 0.27 | 133.55 (113.36, 156.31) |
| Aged 65 plus | 0.28 (0.11, 0.71) | 0.01 | 79.39 (64.00, 97.37) |
| Loess STL | 0.63 (0.53, 0.75) | P<0.001 | 134.70 (127.02, 142.73) |
| Moving average | 0.63 (0.53, 0.75) | P<0.001 | 134.70 (127.02, 142.73) |
| quasi-Poisson | 0.63 (0.52, 0.77) | P<0.001 | 134.70 (127.02, 142.73) |
| 15-day aggregation | 0.63 (0.52, 0.78) | P<0.001 | 134.70 (127.02, 142.73) |
| Gaza war | 0.63 (0.52, 0.78) | P<0.001 | 134.70 (127.02, 142.73) |
| Covid-19 + lockdown | 1.05 (0.74, 1.49) | 0.79 | 134.70 (127.02, 142.73) |
| No Covid-19 cases | 0.63 (0.51, 0.77) | P<0.001 | 135.68 (127.94, 143.77) |

Note. Loess STL and moving averages (MA) are both types of seasonal decompositions. All models, except for “Covid-19 + lockdown,” compute the RR based on dividing the predicted values for the Covid-19 exposure interval with their counterfactual values. The “Covid-19 + lockdown” model computes the RR by dividing the predicted values of Covid-19 + lockdown with the predicted values of Covid-19 alone (with no lockdown).

# eFigure 1 a Basic ITS Design


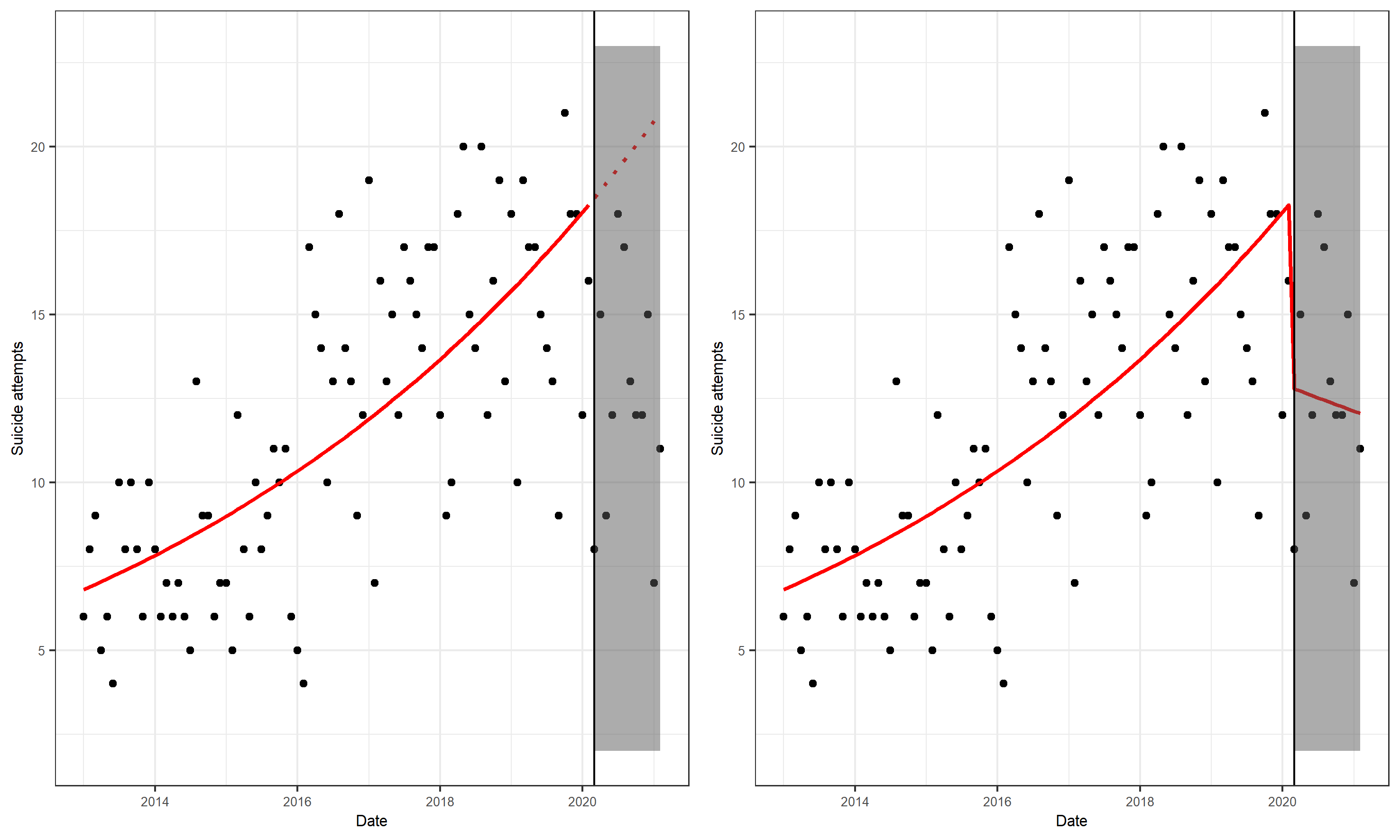


Note. Scatter plot of the monthly count of suicide attempts. White background: pre-Covid-19 period, grey background: post-Covid-19 period. Left: The continuous red line is the fitted pre-Covid-19 trend, and the dotted red line is the predicted trend had Covid-19 not have occurred (couterfactual). Right: The continuous red line is the fitted pre and post Covid-19 pandemic trend.

# eFigure 2 Residual Autocorrelation - Primary Poisson Model


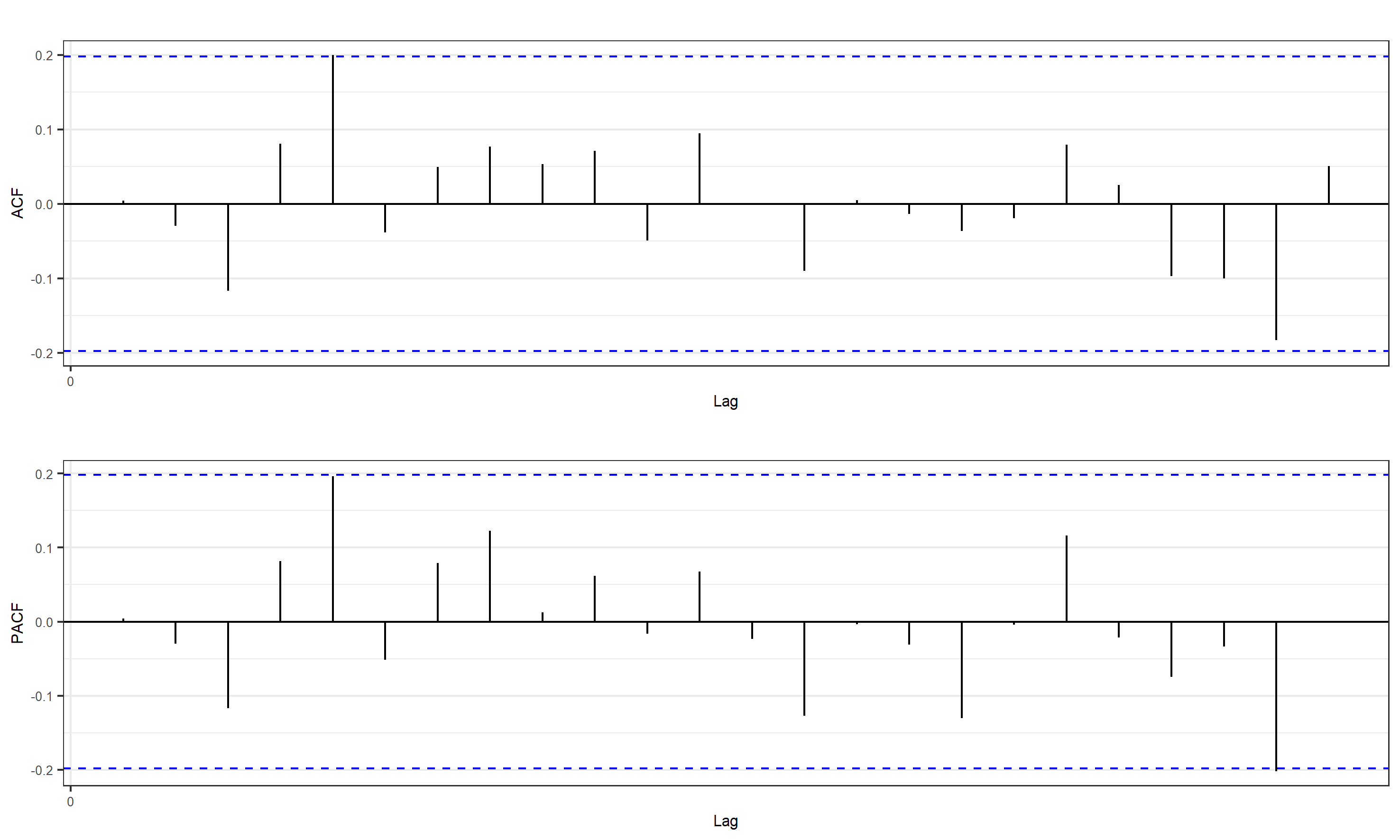


Note. Abbreviations. ACF, autocorrelation function, PACF, partial autocorrelation function. Residual autocorrelation top, residual partial autocorrelation lower panel. The blue dashed lines represent a 95% confidence interval. Any correlation within the blue dashed lines is not considered different than zero. In this case, we observe that all of the ACFs and PACFs are within the confidence interval and hence do not violate the white noise assumption stating the residuals are uncorrelated.

# eFigure 3 Scatterplot and Regression Fitted Values for Males then Females


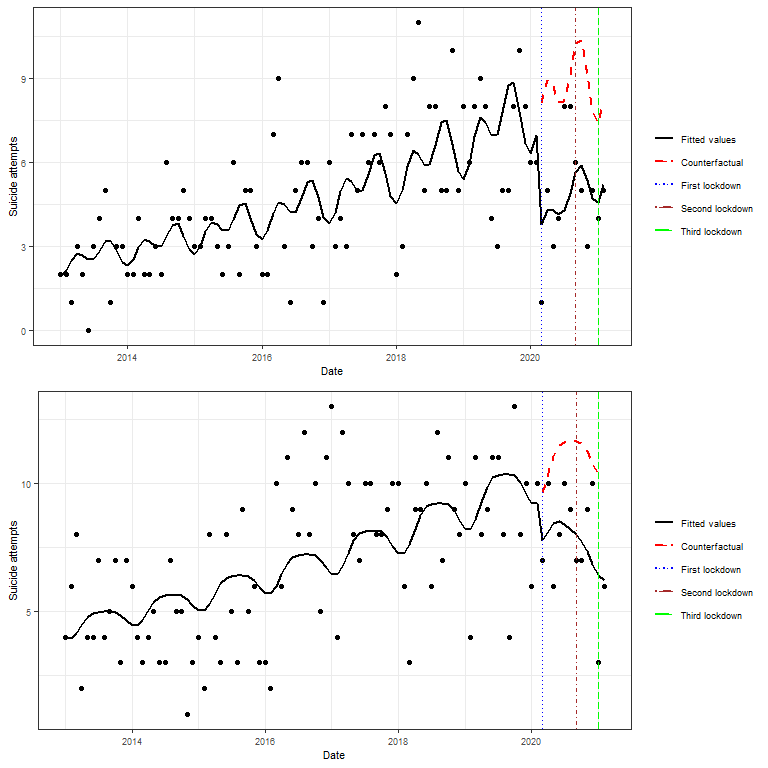


Note. Males top panel, females lower panel. Scatter plot of the monthly count of suicide attempts, together with the regression fitted values (in black), and the counterfactual (in red). The blue horizontal line marks the first lockdown in Israel, the brown horizontal line marks the month of the second lockdown in Israel, and the green horizontal line marks the month of the third lockdown in Israel.

# eFigure 4 Scatterplot and Regression Fitted Values for Males of Working Age and Females of Working Age


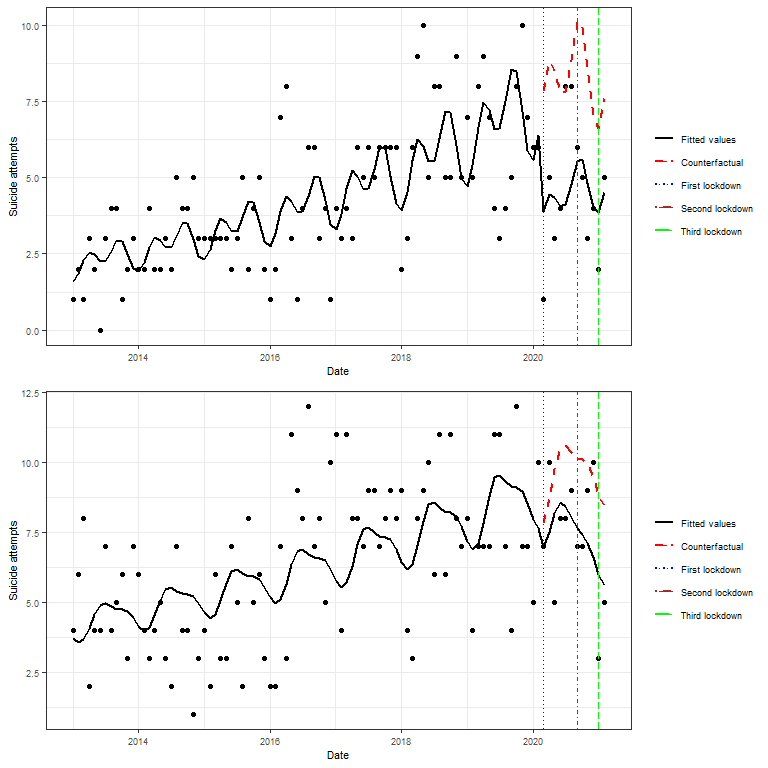


Note. Males of working-age top panel, females of working of age lower panel. Scatter plot of the monthly count of suicide attempts, together with the regression fitted values (in black), and the counterfactual (in red). The blue horizontal line marks the first lockdown in Israel, the brown horizontal line marks the month of the second lockdown in Israel, and the green horizontal line marks the month of the third lockdown in Israel.

# eFigure 5 Scatterplot and Regression Fitted Values for Different SES Groups


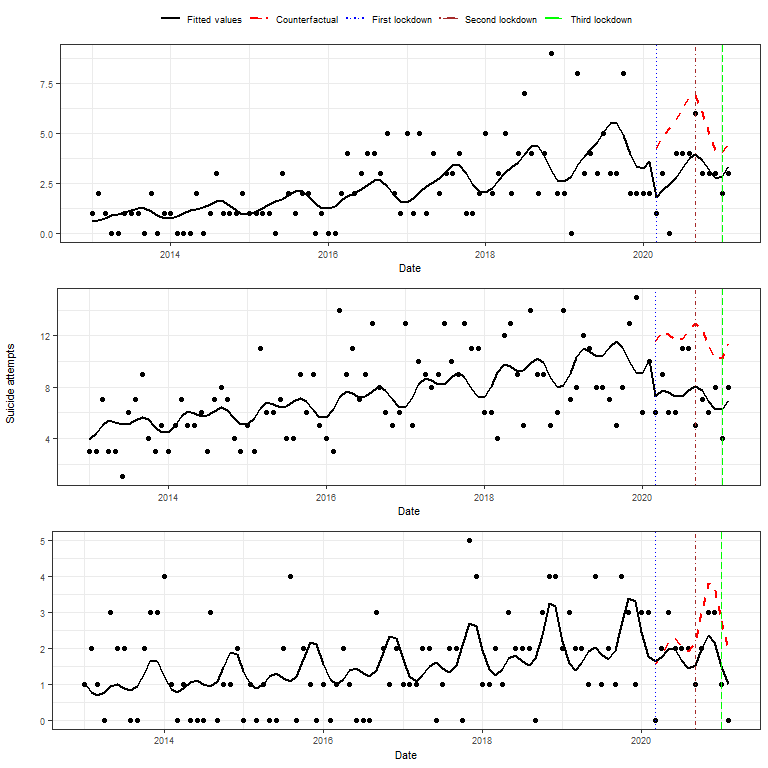


Note. Top low SES, middle medium SES, bottom high SES. Scatter plot of the monthly count of suicide attempts, together with the regression fitted values (in black), and the counterfactual (in red). The blue horizontal line marks the first lockdown in Israel, the brown horizontal line marks the month of the second lockdown in Israel, and the green horizontal line marks the month of the third lockdown in Israel.

# eFigure 6 Scatterplot and Regression Fitted Values for Adults aged over 65


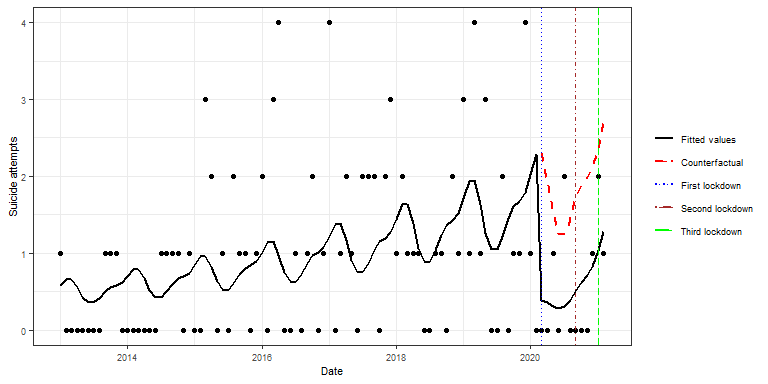
Note. Scatter plot of the monthly count of suicide attempts among the elderly population (over 65), together with the regression fitted values (in black), and the counterfactual (in red). The blue horizontal line marks the first lockdown in Israel, the brown horizontal line marks the month of the second lockdown in Israel, and the green horizontal line marks the month of the third lockdown in Israel.

# eFigure 7 Scatterplot and Regression Fitted Values for STL and MA Seasonal Decompositions


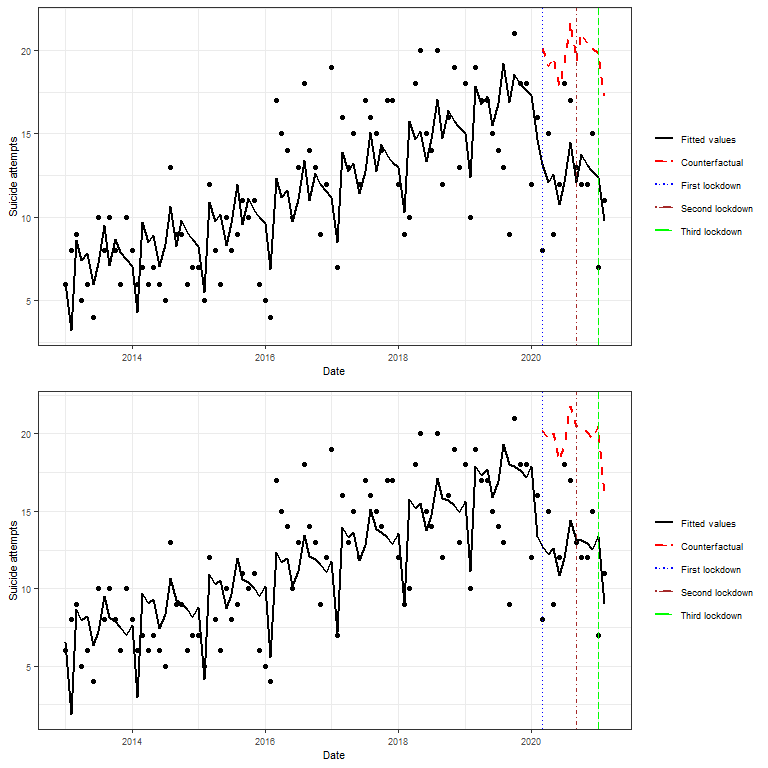


Note. STL seasonal decomposition top, MA seasonal decomposition lower panel. Scatter plot of the monthly count of suicide attempts, together with the regression fitted values (in black), and the counterfactual (in red). The blue horizontal line marks the first lockdown in Israel, the brown horizontal line marks the month of the second lockdown in Israel, and the green horizontal line marks the month of the third lockdown in Israel.

# eFigure 8 Scatterplot and Regression Fitted Values for Quasi-Poisson Regression


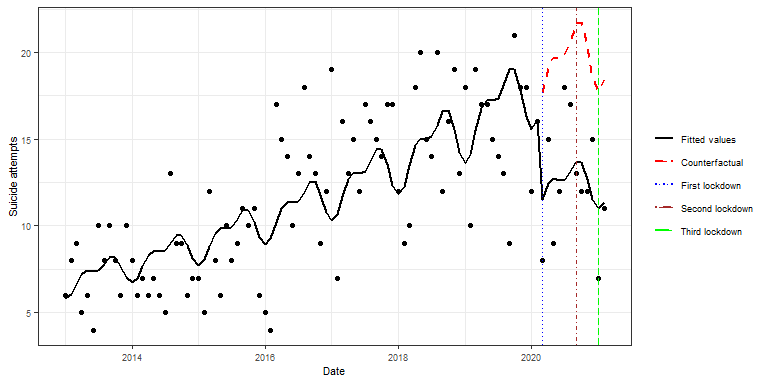


Note. Scatter plot of the monthly count of suicide attempts, together with the regression fitted values (in black), and the counterfactual (in red). The blue horizontal line marks the first lockdown in Israel, the brown horizontal line marks the month of the second lockdown in Israel, and the green horizontal line marks the month of the third lockdown in Israel.

# eFigure 9 Scatterplot and Regression Fitted Values for 15 Day Intervals


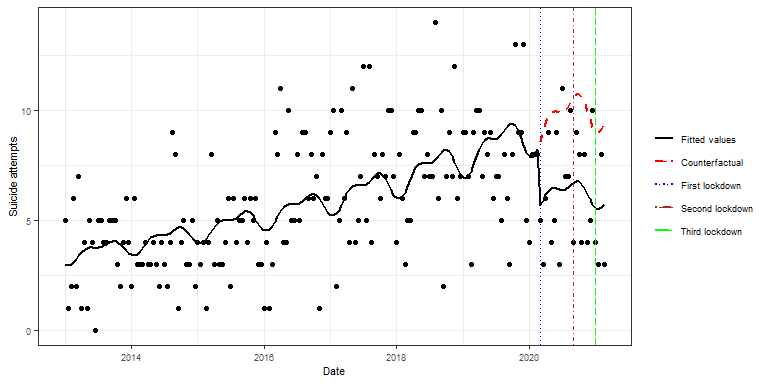


Note. The scatter plot shows the biweekly count of suicide attempts, together with the regression fitted values (in black), and the counterfactual (in red). The blue horizontal line marks the first lockdown in Israel, the brown horizontal line marks the month of the second lockdown in Israel, and the green horizontal line marks the month of the third lockdown in Israel.

# eFigure 10 Scatterplot and Regression Fitted Values for Covid-19 and 2014 Gaza War


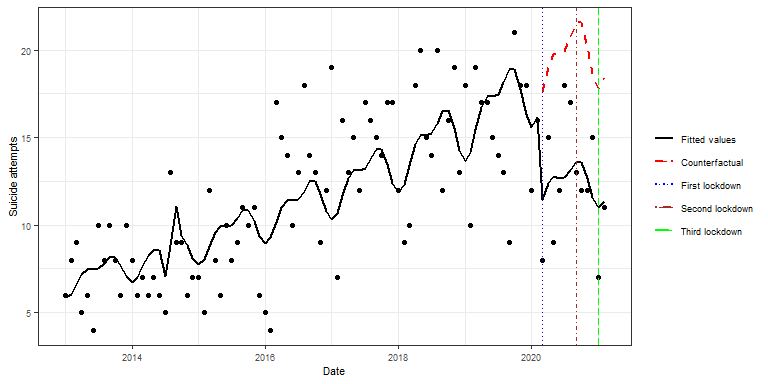


Note. Scatter plot of the monthly count of suicide attempts, together with the regression fitted values (in black), and the counterfactual (in red). The blue horizontal line marks the first lockdown in Israel, the brown horizontal line marks the month of the second lockdown in Israel, and the green horizontal line marks the month of the third lockdown in Israel.

# eFigure 11 Scatterplot and Regression Fitted Values for 15 Day Intervals and Covid-19 + Lockdown


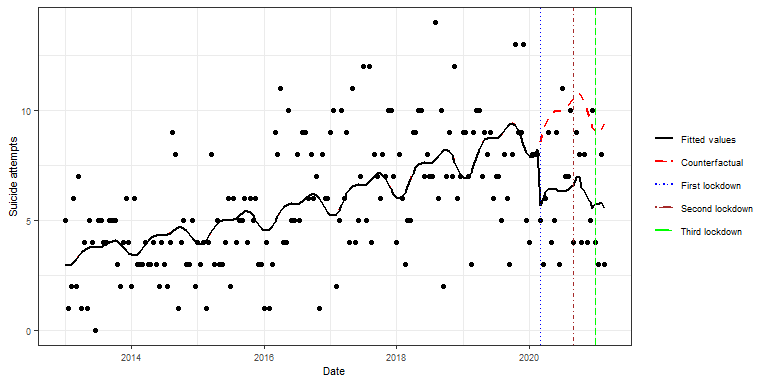


Note. The scatter plot shows the biweekly count of suicide attempts, together with the regression fitted values (in black), and the counterfactual (in red). The blue horizontal line marks the first lockdown in Israel, the brown horizontal line marks the month of the second lockdown in Israel, and the green horizontal line marks the month of the third lockdown in Israel.

# eFigure 12 Scatterplot and Regression Fitted Values restricted to non Covid-19 Cases


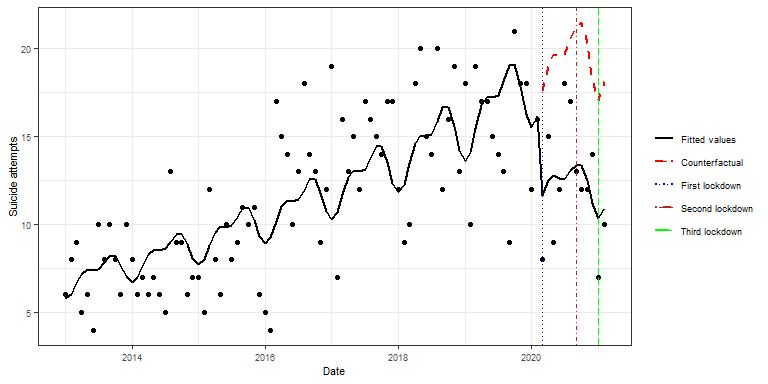


Note. Scatter plot of the monthly count of suicide attempts, together with the regression fitted values (in black), and the counterfactual (in red). The blue horizontal line marks the first lockdown in Israel, the brown horizontal line marks the month of the second lockdown in Israel, and the green horizontal line marks the month of the third lockdown in Israel.

# eReferences

Central Bureau of Statistics. (1995). *Demographic Characteristics of the Population in Localities and Statistical Areas*. Jerusalem, Israel

Goldberg, S., Fruchter, E., Davidson, M., Reichenberg, A., Yoffe, R., & Weiser, M. (2011). The relationship between risk of hospitalization for schizophrenia, SES, and cognitive functioning. *Schizophrenia Bulletin*, *37*(4), 664-670. https://doi.org/10.1093/schbul/sbr047

Hale, T., Angrist, N., Goldszmidt, R., Kira, B., Petherick, A., Phillips, T., . . . Tatlow, H. (2021). A global panel database of pandemic policies (Oxford COVID-19 Government Response Tracker). *Nature Human Behavior*, *5*(4), 529-538. https://doi.org/10.1038/s41562-021-01079-8

Hyndman, R. J., & Khandakar, Y. (2008). Automatic Time Series Forecasting: The forecast Package for R. *Journal of Statistical Software*, 27(3), 1–22. https://doi.org/10.18637/jss.v027.i03

Levine, S. Z., Levav, I., Goldberg, Y., Pugachova, I., Becher, Y., & Yoffe, R. (2016). Exposure to genocide and the risk of schizophrenia: a population-based study. *Psychological Medicine*, *46*(4), 855-863. https://doi.org/10.1017/S0033291715002354

Levine, S. Z., Levav, I., Yoffe, R., Becher, Y., & Pugachova, I. (2016). Genocide Exposure and Subsequent Suicide Risk: A Population-Based Study. *PLoS One*, *11*(2), e0149524. https://doi.org/10.1371/journal.pone.0149524
